# Quantitative evaluation of the subsequent hemorrhage with arteriography-derived hemodynamic features in patients with untreated cerebral arteriovenous malformation

**DOI:** 10.3389/fneur.2023.1174245

**Published:** 2023-08-16

**Authors:** Haoyu Zhu, Lian Liu, Yuzhou Chang, Yuqi Song, Shikai Liang, Chao Ma, Longhui Zhang, Fei Liang, Chuhan Jiang, Yupeng Zhang

**Affiliations:** ^1^Department of Neurosurgery, Beijing Neurosurgical Institute and Beijing Tiantan Hospital, Capital Medical University, Beijing, China; ^2^Department of Neurosurgery, School of Clinical Medicine, Beijing Tsinghua Changgung Hospital, Tsinghua University, Beijing, China; ^3^Department of Interventional Radiology and Vascular Surgery, Peking University Third Hospital, Beijing, China

**Keywords:** arteriovenous malformation, hemodynamics, subsequent hemorrhage, time density curve, angiographic parametric imaging

## Abstract

**Background:**

Patients with untreated cerebral arteriovenous malformations (AVMs) are at risk of intracerebral hemorrhage. However, treatment to prevent AVM hemorrhage carries risks.

**Objective:**

This study aimed to analyze the AVM nidus-related hemodynamic features and identify the risk factors for subsequent hemorrhage.

**Methods:**

We retrospectively identified patients with untreated AVMs who were assessed at our institution between March 2010 and March 2021. Patients with ≥6 months of treatment-free and hemorrhage-free follow-up after diagnosed by digital subtraction angiography were included in subsequent examinations. The hemodynamic features were extracted from five contrast flow-related parameter maps. The Kaplan-Meier analyses and Cox proportional hazards regression models were used to find the potential risk factors for subsequent hemorrhage.

**Results:**

Overall, 104 patients with a mean follow-up duration of 3.37 years (median, 2.42 years; range, 6–117 months) were included in study, and the annual risk of rupture was 3.7%. Previous rupture (hazard ratio [HR], 4.89; 95% confidence interval [CI], 1.16–20.72), deep AVM location (HR, 4.02; 95% CI, 1.01–15.99), higher cerebral blood volume (HR, 3.35; 95% CI, 1.15–9.74) in the nidus, and higher stasis index (HR, 1.54; 95% CI, 1.06–2.24) in the nidus were associated with subsequent hemorrhage in untreated AVMs.

**Conclusion:**

Higher cerebral blood volume and stasis index in the nidus suggest increased blood inflow and stagnant blood drainage. The combination of these factors may cause subsequent hemorrhage of AVMs.

## Introduction

Intracerebral hemorrhage (ICH) is one of the most typical presentations of cerebral arteriovenous malformations (AVMs). Hemorrhagic stroke caused by ICH is linked to mortality and long-term neurological impairment (1). The overall annual risk of rupture in all types of AVMs in previous studies was 2–4% (2, 3), typically affecting the younger population (4). The current treatment therapies for AVMs include endovascular and surgical treatments. Nevertheless, owing to the complicated cerebrovascular architecture of AVMs, inadequate therapy carries an even greater risk of complications than spontaneous rupture (5). Consequently, to achieve potential benefits from a risky treatment, clinicians need to carefully screen for high-rupture-risk AVMs.

Over the past several decades, numerous angioarchitecture characteristics, including previous hemorrhagic status, AVM location, venous drainage pattern, associated aneurysms, and nidus size, have been reported as potential risk factors for subsequent hemorrhage (6–11). Besides traditional demographic and angioarchitecture characteristics, hemodynamic features are also important in the evaluation of AVM rupture risk. Several studies have attempted to examine the hemodynamics of AVM, but their findings were focused on regions of interest (ROI) as opposed to pixel-by-pixel analysis. Furthermore, their ROIs were mainly limited to the draining vein or feeding artery, not to the AVM nidus itself (12–14). Angiographic parametric imaging (API) can synthesize the time–density curve (TDC) of ROIs and therefore calculate quantitative hemodynamic parameters (15). In the current study, we used this technology to quantitatively evaluate the hemodynamic features of the AVM nidus and to examine the connection between these features and subsequent hemorrhage in untreated AVMs.

## Materials and methods

### Study design

This study retrospectively identified consecutive patients of all ages with untreated AVM admitted to our institution between March 2010 and March 2021. The inclusion criteria were: (1) patients with an untreated AVM diagnosed by digital subtraction angiography (DSA) at our institution, and (2) patients with ≥6 months of treatment-free and hemorrhage-free follow-up after diagnosed DSA. The exclusion criteria were: (1) pial arteriovenous fistula, spinal AVM, or any other type of cerebral vascular malformations, (2) the quality of DSA data was to poor for further analysis, and (3) patients missing the baseline information. This study was approved by the ethical committee of Beijing Tiantan Hospital and the written informed consent was provided by all patients.

If an AVM showed signs of hemorrhage on computed tomography (CT) and was consistent with a history of rupture, it was assumed to have ruptured before admission. Demographic information, including age and sex, was collected from medical records. The pediatric patient population was defined as individuals aged <18 years. AVM-related characteristics data, including the number of feeding arteries, nidus size, AVM location, the presence of AVM-related aneurysms, and the patterns of venous drainage, were obtained. AVM size, feeding artery, and venous drainage were divided into two categories: small (<3 cm) or large (≥3 cm), single (*n* = 1) or multiple (*n* ≥ 2), and exclusively deep or others, respectively. If the lesion involved the cerebellum, basal ganglia, brain stem, or thalamus, the location of AVM was considered deep; otherwise, it was considered superficial.

The clinical characteristics and AVM-related hemodynamic features were collected and analyzed from the time of initial AVM diagnosis using DSA at our institution. Following the initial DSA, patients were followed until one of the following occurred: (1) the first subsequent hemorrhage, (2) any treatment for AVM, (3) death, or (4) the end of March 2021. The primary endpoint of our study was AVM-related ICH after initial DSA diagnosis at our institution. During the follow-up period, the definition of subsequent hemorrhage of AVMs was a symptomatic clinical occurrence with fresh cerebral blood signs on CT or magnetic resonance imaging, with no other cause that could be readily identified as more probable than AVMs. By dividing the number of subsequent rupture events by the total person-years of follow-up, the annual risk of AVMs subsequent hemorrhage was calculated.

Conservative treatment methods were selected for the following reasons: (1) the AVM was considered to have a low risk of rupture and the patient was asymptomatic; (2) using present techniques, the risks of treatment for AVMs were disproportionate to the benefits, or (3) the patient or family refused to accept the risks associated with any treatment option.

### DSA image acquisition and ROI delineation

DSA images were acquired on the 722038-153 Station (Philips, Netherlands) and AW6302 Station (GE, United States). The DSA acquisition procedure consisted of four frames per second. For each DSA operation, the contrast agent was injected at either the cervical section of the internal carotid artery at 4 mL/s for 6 mL or the vertebral artery at 3 mL/s for 5 mL. The DSA images that best depicted the nidus were chosen for further analysis.

**Figure 1:**
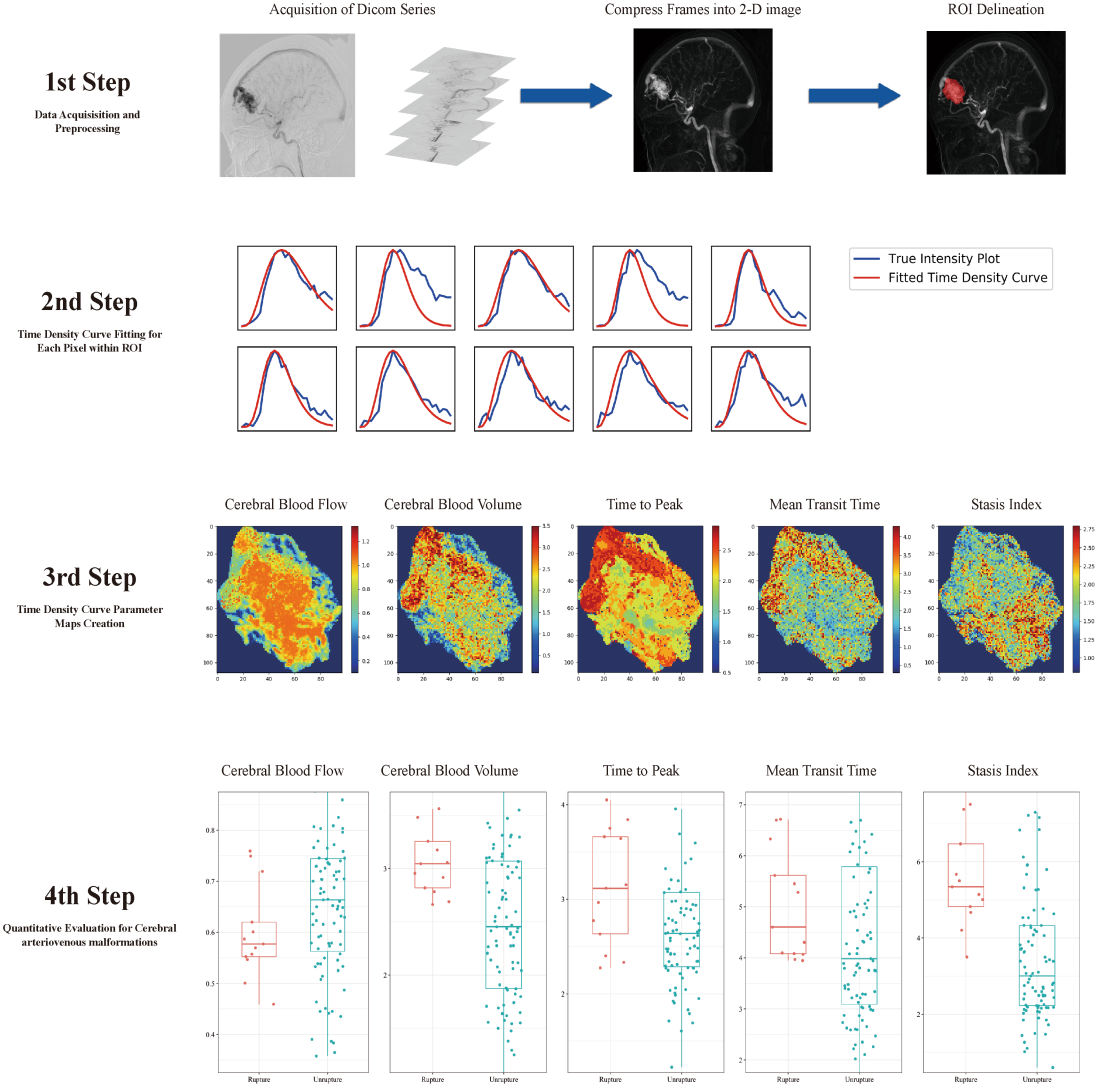
Model development flowchart. In step 1, a two-dimensional map is created, and the region of interest (ROI) is delineated. In step 2, the time–density curve (TDC) for each pixel of the ROI is calculated. In step 3, five parameter maps are created according to the TDC. In step 4, quantitative hemodynamic features are extracted from the TDC parameter maps.

To quantitatively analyze the AVM nidus, two interventional neuroradiologists who were blinded to the patient information delineated the ROI for the quantitative assessment of the AVM, including the nidus and reference ROIs, on the two-dimensional map (Figure 1).

### TDC fitting, TDC-based parameter map calculation, and AVM quantitative evaluation

TDC was fitted using a simplified gamma-variate function (16). As described in our earlier publication (17), the image preparation and TDC fitting programs were developed in Python (3.6.1). Each parameter map was generated by calculating the respective characteristics of the TDC at each pixel in the selected ROI (Figure 1).

For each selected DSA series, five contrast flow-related parameter maps were created by calculating the TDC at each pixel of the selected ROI. Time to peak (TTP) was defined as the time taken by the TDC to reach its highest value; mean transit time (MTT) was determined by calculating the average residence duration of the contrast medium; cerebral blood volume (CBV) is a relative blood flow volume index that was obtained by integrating the TDC over 6 s; cerebral blood flow (CBF) was determined by dividing CBV by MTT. The inflow gradient and outflow gradient were defined as the steepest and shallowest slopes in all linear functions that were fitted in the gamma variant function, and the stasis index was defined as dividing the inflow gradient by the absolute value of the outflow gradient. The ultimate parameters were determined by calculating the average gray level intensity within the five TDC-based parameter maps, and the following characteristics were used for the hemodynamic analysis of the nidus: CBF, CBV, TTP, MTT, and stasis index.

### Statistical analyses

The statistical analyses were conducted using the R software (version 4.0.1). Continuous variables are presented as mean and standard deviation for descriptive statistics, whereas categorical variables are provided as frequency and percentage. For continuous variables, the difference between groups was calculated using Student’s *t*-test or Wilcoxon’s rank-sum test, as appropriate, after assessing normality using the Shapiro–Wilk test. Categorical variables were compared using the chi-square test. Cox proportional hazards analysis was performed to examine the relevance of selected variables in predicting the relative risk of time from diagnosis to first subsequent hemorrhage, any treatment for AVM, death, or end of follow-up (March 2021). Variables that yielded a *p* value of less than 0.05 in the univariate Cox regression were subsequently incorporated into the multivariate Cox regression analysis. The Kaplan–Meier model was used to illustrate the rate of hemorrhage-free survival for untreated patients with and without specific features.

## Results

### Demographics and clinical characteristics

One hundred and four consecutive patients with untreated AVMs and with ≥6 months of hemorrhage-free and treatment-free follow-up were included. Among them, 30 (28.8%) patients were aged <18 years, with the average age being 12.7 years. The whole cohort had a mean age of 29.0 ± 14.3 years at initial diagnosis. There were 51 and 53 patients with small (<3 cm) and large (≥3 cm) nidus sizes, respectively. The temporal lobe was the most frequent AVM location (23.1%). Overall, 19.2% (20/104) of AVMs had exclusively deep venous drainage, whereas 28.8% (30/104) had a single feeding artery. Eight patients presented with intranidal aneurysms. Finally, 85 patients had Spetzler–Martin grade I, II, or III AVM and 19 patients had grade IV or V AVM.

### Hemorrhage presentation

In this cohort, the incidence of hemorrhagic presentation was 38.5% (40/104). Children (<18 years) had a significantly higher proportion of hemorrhage at presentations in this series (*p* = 0.015). The proportion of AVMs with a small size (*p* = 0.001), single feeding artery (*p* = 0.015), or exclusively deep venous drainage pattern (*p* = 0.001) was significantly higher among patients with hemorrhage at presentation. In this study, we found no statistically significant differences in the five hemodynamic parameters between AVMs that had hemorrhage at presentation and those that had not previously ruptured. Table 1 summarizes the baseline characteristics.

**Table 1:** Comparison of baseline characteristics and clinical features of patients with ruptured and unruptured arteriovenous malformation (AVMs) at initial diagnosis.

Parameter	All patients (*n* = 104)	Ruptured AVMs (*n* = 40)	Unruptured AVMs (*n* = 64)	*p*-value
Age at admission (years), mean (standard deviation)	29.0 ± 14.3	25.7 ± 14.2	31.1 ± 14.1	0.070
**Age group**				0.015
Childhood	30 (28.8%)	17 (42.5%)	13 (20.3%)	
Adult	74 (71.2%)	23 (57.5%)	51 (79.7%)	
**Sex**				0.877
Male	51 (49.0%)	20 (50.0%)	31 (48.4%)	
Female	53 (51.0%)	20 (50.0%)	33 (51.6%)	
**Nidus size**				0.001
Small (0–3 cm)	51 (49.0%)	30 (75.0%)	21 (32.8%)	
Large (>3 cm)	53 (51.0%)	10 (25.0%)	43 (67.2%)	
**Location**				0.479
Temporal lobe	24 (23.1%)	7 (17.5%)	17 (26.6%)	
Occipital lobe	21 (20.2%)	8 (20.0%)	13 (20.3%)	
Parietal lobe	13 (12.5%)	3 (7.5%)	10 (15.6%)	
Frontal lobe	19 (18.3%)	8 (20.0%)	11 (17.2%)	
Cerebellum	7 (6.7%)	2 (5.0%)	5 (7.8%)	
Basal ganglia	8 (7.7%)	5 (12.5%)	3 (4.7%)	
Brain stem	5 (4.8%)	3 (7.5%)	2 (3.1%)	
Thalamus	7 (6.7%)	4 (10.0%)	3 (4.7%)	
**No. of feeding arteries**				0.015
Single feeding artery	30 (28.8%)	17 (42.5%)	13 (20.3%)	
Multiple feeding arteries	74 (71.2%)	23 (57.5%)	51 (79.7%)	
**Venous drainage pattern**				0.001
Exclusively deep	20 (19.2%)	14 (35.0%)	6 (9.4%)	
Superficial or both deep and superficial	84 (80.8%)	26 (65.0%)	58 (90.6%)	
**Intranidal aneurysm**				0.749
Yes	8 (7.7%)	4 (10.0%)	4 (6.2%)	
No	96 (92.3%)	36 (90.0%)	60 (93.8%)	
**Spetzler–Martin grade**				0.718
I	18 (17.3%)	9 (22.5%)	9 (14.1%)	
II	35 (33.7%)	12 (30.0%)	23 (35.9%)	
III	32 (30.8%)	12 (30.0%)	20 (31.3%)	
IV	16 (15.4%)	6 (15.0%)	10 (15.6%)	
V	3 (2.9%)	1 (2.5%)	2 (3.1%)	
**Follow-up**				0.572
Total person-years	350.4	123.9	226.5	
Median (range), years	2.42 (0.5–9.8)	1.75 (0.5–9)	2.79 (0.5–9.8)	
**Quantitative features**				
CBV, IS	2.57 ± 0.78	2.46 ± 0.84	2.64 ± 0.74	0.291
CBF, I	0.65 ± 0.15	0.65 ± 0.16	0.66 ± 0.15	0.891
MTT, S	4.67 ± 2.01	4.31 ± 1.79	4.89 ± 2.12	0.369
TTP, S	2.85 ± 0.88	2.80 ± 0.91	2.89 ± 0.87	0.833
Stasis index	3.79 ± 1.88	3.96 ± 1.92	3.68 ± 1.86	0.348

CBV, cerebral blood volume; CBF, cerebral blood flow; MTT, mean transit time; TTP, time to peak; I, intensity; S, second.

### Follow-up outcomes

The mean follow-up period was 3.37 years (median, 2.42 years; range, 6–117 months). During the entire 350.4 person-years between initial diagnostic DSA and the follow-up endpoint, a total of 13 patients experienced subsequent hemorrhage of AVMs, resulting in an annual rupture rate of 3.7% for this whole cohort. Among the 40 patients with AVMs that have hemorrhage at presentation, the mean follow-up period was 3.10 years (median, 1.75 years; range, 6–108 months). During this follow-up period, 9 out of these 40 patients experienced subsequent hemorrhagic events, corresponding to an annual rupture risk of 7.3%. Conversely, for the remaining 64 patients who initially presented with unruptured AVMs, the mean follow-up duration was 3.54 years (median, 2.79 years; range, 6–117 months). During this period, 4 patients experienced subsequent hemorrhagic events, reflecting an annual rupture risk of 1.8%. Six patients died throughout the follow-up period. Most of their deaths, except the one from congenital heart disease, were thought to be mostly attributable to AVM.

**Figure 2:**
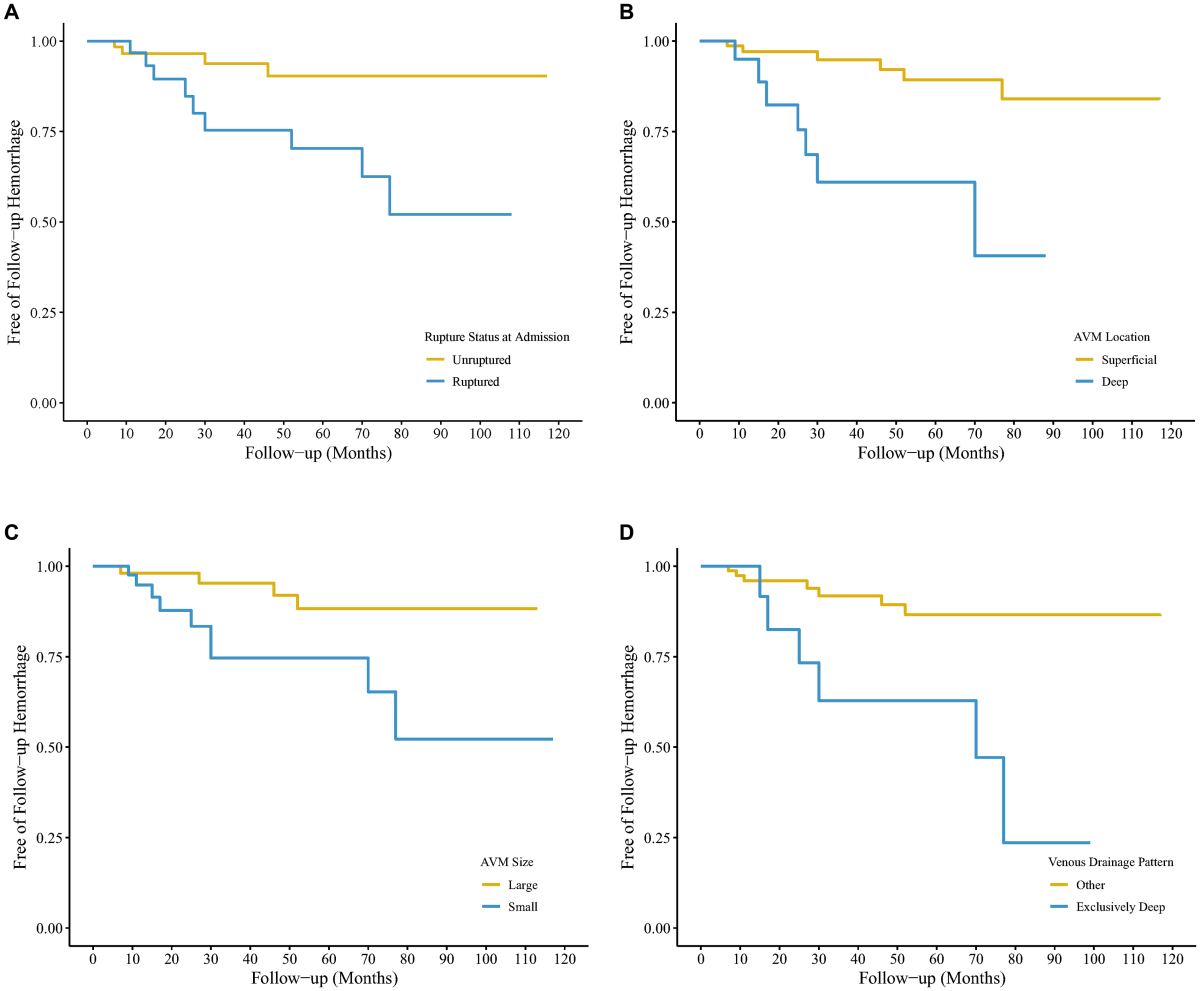
Kaplan–Meier survival curves of hemorrhage-free survival after the initial arteriovenous malformation (AVM) diagnosis using digital subtraction angiography (DSA) for 104 untreated patients. The four survival graphs are based on model estimates for **(A)** hemorrhagic vs. non-hemorrhagic AVM presentation, **(B)** Deep vs. superficial AVM location, **(C)** small vs. larger AVM size, and **(D)** other vs. exclusively deep venous drainage pattern.

**Figure 3:**
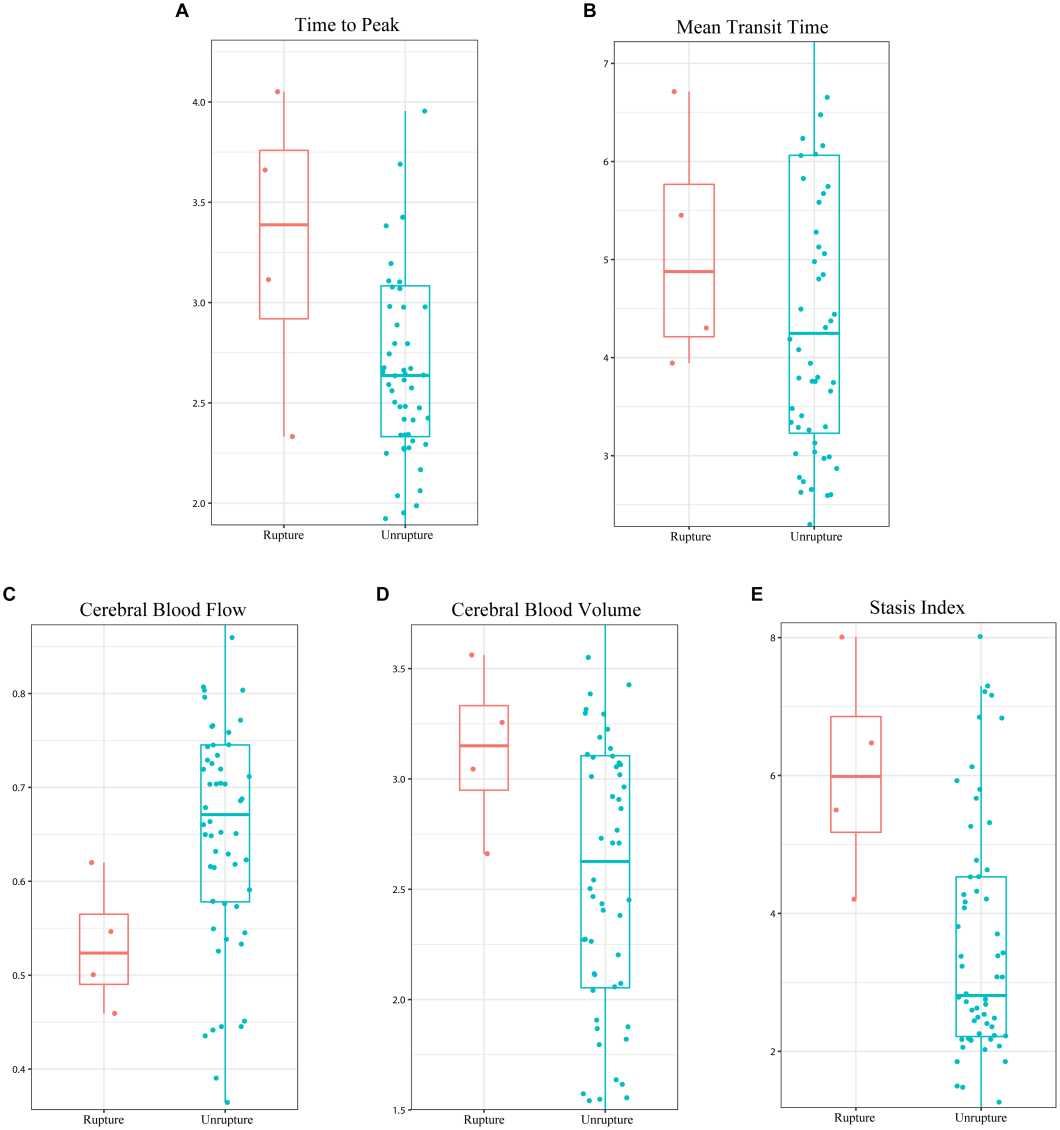
Boxplot illustrating the difference in the five hemodynamic features between the subsequent hemorrhage and hemorrhage-free in unruptured arteriovenous malformation. **(A)** Time to peak, **(B)** mean transit time, **(C)** cerebral blood flow, **(D)** cerebral blood volume, and **(E)** stasis index.

As shown in Figure 2, previous rupture and deep location significantly enhanced the probability of AVM-related subsequent hemorrhage. However, although small nidus size and exclusively deep venous drainage pattern increased AVM-related subsequent hemorrhage risk, the differences were not significant in the log-rank test. Figure 3 shows the distribution of five hemodynamic features in patients with and without subsequent hemorrhage, only one feature—CBF—had a larger median value in patients with subsequent AVM rupture.

**Figure 4:**
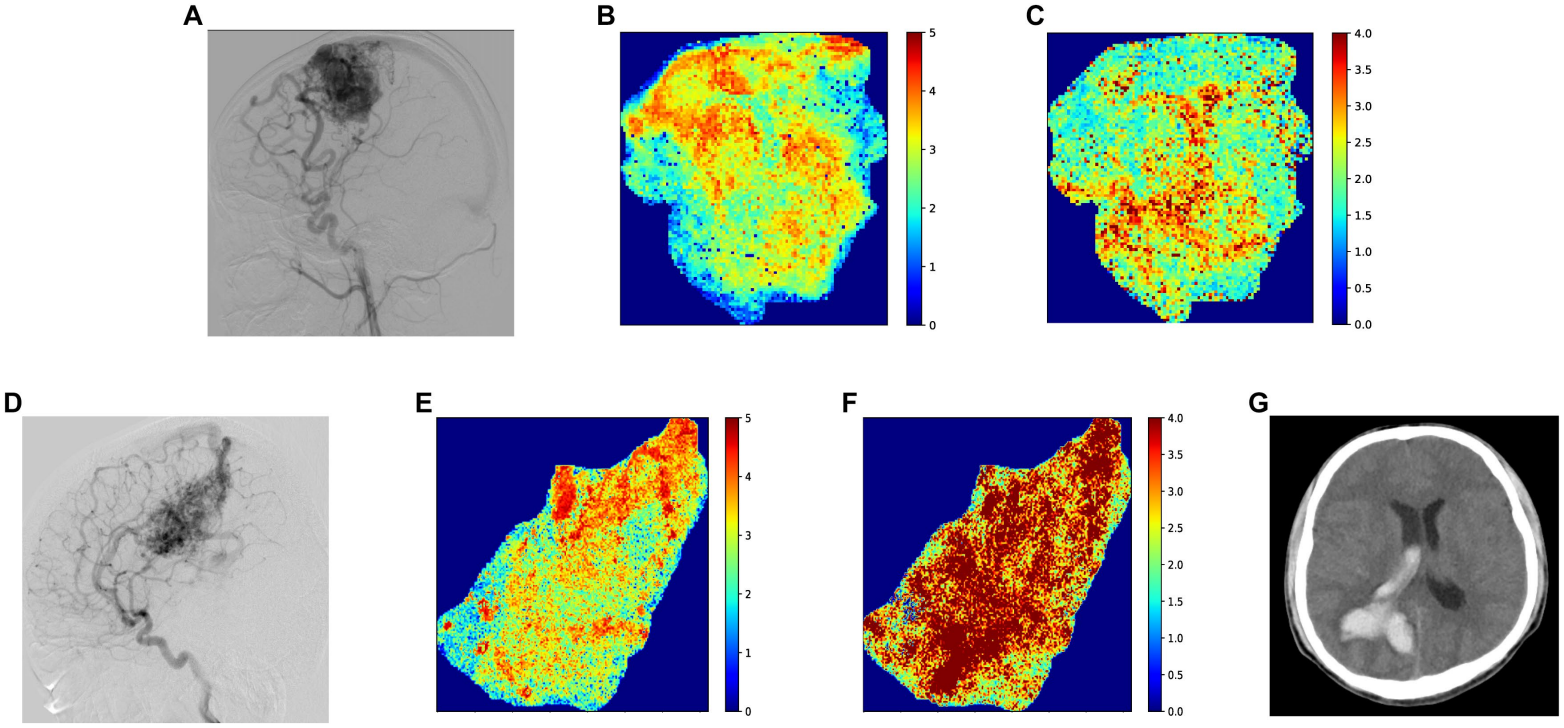
**(A)** Standard lateral digital subtraction angiography (DSA) image of a patient in their 20 s at the time of diagnosis. After this DSA, the patient received conservative treatment for 14 months without subsequent rupture, before undergoing gamma knife therapy. **(B)** Color-coded image of cerebral blood volume (CBV) parameters, with the patient’s CBV at 2.41 IS (intensity*second) based on the average gray level intensity within the map. **(C)** Color-coded image depicting the stasis index of the nidus, which shows a stasis index of 3.64. **(D)** Standard lateral DSA image of a patient in their 20 s at diagnosis, who experienced a subsequent AVM hemorrhage 30 months after this DSA. **(E)** Color-coded image illustrating CBV parameters, with a quantitative result of 3.56 IS based on the average gray level intensity. **(F)** Color-coded image presenting the stasis index for the nidus, demonstrating a stasis index of 6.47. **(G)** Thirty months after the initial diagnostic DSA, intracerebral hemorrhage was identified on computed tomography, with no other more probable causes than AVM readily identifiable.

Univariate risk analysis showed associations between ruptured AVM (*p* = 0.021), nidus size (*p* = 0.026), AVM location (*p* = 0.003), venous drainage pattern (*p* = 0.004), CBV of the nidus (*p* = 0.016), stasis index (*p* = 0.001), and subsequent hemorrhage. The effects of sex, age, number of feeding arteries, intranidal aneurysm, CBF, MTT, and TTP were not significant. In the multivariate Cox proportional hazards model, previous rupture (hazard ratio [HR], 4.89; 95% confidence interval [CI], 1.16–20.72), deep location (HR, 4.02; 95% CI, 1.01–15.99), higher CBV (HR, 3.35; 95% CI, 1.15–9.74), and higher stasis index (HR, 1.54; 95% CI, 1.06–2.24) were independent risk factors for subsequent rupture (Table 2). Figure 4 illustrated two patients’ contrast flow-related parameter maps of CBV and stasis index.

**Table 2:** Univariate and multivariate Cox proportional hazards models for risk of subsequent hemorrhage of 104 untreated arteriovenous malformation (AVMs) during the follow-up period.

Parameters	Univariate			Multivariate		
	Hazard ratio	95% CI	*p*-value	Hazard ratio	95% CI	*p*-value
Female sex	1.69	0.55–5.16	0.360			
Pediatric patients	1.09	0.33–3.53	0.892			
Previous rupture	4.02	1.23–13.06	0.021	4.89	1.16–20.72	0.031
Small nidus size	3.83	1.17–12.53	0.026	1.71	0.36–8.14	0.502
Deep AVM location	5.53	1.82–16.83	0.003	4.02	1.01–15.99	0.049
Single feeding arteries	1.84	0.60–5.64	0.286			
Exclusively deep drainage	5.04	1.69–15.05	0.004	1.23	0.24–6.33	0.804
Intranidal aneurysm	3.14	0.86–11.45	0.083			
**Quantitative features**						
CBV, IS	2.52	1.19–5.35	0.016	3.35	1.15–9.74	0.027
CBF, I	0.12	0.01–3.98	0.238			
MTT, S	1.06	0.83–1.35	0.639			
TTP, S	1.38	0.80–2.35	0.245			
Stasis index	1.48	1.16–1.88	0.001	1.54	1.06–2.24	0.022

Pediatric patients: aged < 18 years. CBV, cerebral blood volume; CBF, cerebral blood flow; MTT, mean transit time; TTP, time to peak; I, intensity; S, second.

## Discussion

This study quantitatively evaluated the hemodynamic characteristics of the AVM nidus and revealed that quantitative hemodynamic features could be used as a potential indicator to predict subsequent hemorrhage. Our results demonstrated that AVM with hemorrhagic presentation, deep location, higher CBV, and stasis index in the nidus were associated with a higher risk of subsequent hemorrhage.

Several publications on the risk assessment of treatment benefits for AVM have been reported. The ARUBA study indicated that the risk of death or symptomatic stroke in patients with unruptured AVM due to inappropriate treatment was higher than that due to medical management alone (5, 18). However, while hesitancy to receive high-risk interventional therapy is understandable, this risk should be weighed against that of subsequent hemorrhage without treatment. Therefore, evaluating and predicting the risk of subsequent hemorrhage might help weigh the risks of conservative treatment and interventional therapy for AVM. Feghali et al. (19) suggested a unique score for AVM that took into account risk factors including nonwhite race, small nidus size, deep location, single feeding artery, and seclusive deep venous drainage. However, the area under the curve was only 0.698 in the validation data set. Therefore, besides clinical features, there may still be additional risk factors that have not yet been identified. Raoult et al. (20) quantitatively examined AVM-related hemodynamic characteristics and reported that a higher rupture risk of AVM was associated with a lower venous-to-arterial time-to-peak ratio. Chen et al. (14) explored the potential risk factors for unruptured AVMs and found that increased blood flow and a shorter MTT across the AVMs nidus were potential risk factors for silent intralesional microhemorrhages. Although both of their studies identified quantitative hemodynamic factors associated with AVM hemorrhage, their measurement was still indirect because the ROIs used for calculations did not involve the nidus. Furthermore, rather than looking at AVMs that were about to rupture, most previous research looked at ruptured AVMs to determine the risk variables for AVM hemorrhage. As demonstrated in the study by Jin et al. (21) that stated that approximately 40% of ruptured AVMs had angioarchitecture changes, there may be some limitations on the evaluation of a ruptured AVM to determine the probability of subsequent hemorrhage.

By iteratively calculating the TDC acquired from quantitative DSA, Lin et al. (13) first proposed the stasis index, and they discovered a correlation between AVM rupture and a high stasis index of the main drainage veins. Their investigation evaluated the hemodynamic state of the feeding artery and venous drainage portion of the AVM. A recent publication reported by Chen et al. (22) found that a higher stasis index and slower transmittal relative velocity were effective indicators of subsequent hemorrhage of AVMs. Based on these studies, the present study further validated the stasis index by analyzing the whole nidus pixel-by-pixel and found that a higher stasis index and CBV in the nidus were associated with subsequent hemorrhage. This indicates that there was increased blood inflow and stagnant blood drainage, which may be the cause of the subsequent hemorrhage of AVMs.

Besides hemodynamic features, hemorrhagic presentation and deep location turned out to be significant independent predictors of subsequent hemorrhage, with approximately five-fold and four-fold risks compared with those without subsequent hemorrhage, respectively. Concerning the effect of hemorrhagic presentation, many studies have demonstrated that previous ruptured AVMs were more prone to undergo a subsequent hemorrhage (3, 8, 9, 23–25). Yamada et al. (9) discovered that the annual rupture risk for patients with unruptured AVMs was 3.1%, whereas patients with previously ruptured AVMs faced a higher annual rupture risk of 6.8%. The findings reported by Yamada et al. (9) align closely with the observations made in the present study that the previously ruptured AVM were more prone to undergo a subsequent hemorrhage. Tong et al. (25) reported 149 patients with untreated AVMs and discovered that a prior rupture may only significantly raise the risk of subsequent rupture in initial 5 years. Due to the relatively short follow-up period, the present study did not investigate the evolution of subsequent rupture risk over time. Several studies have reported that the deep location of AVMs may be one of the risk factors for subsequent hemorrhage (3, 8, 9). In a recent publication by Chen et al. (26), the authors developed and demonstrated the efficacy of a VALE scoring system for assessing hemorrhage risk in AVM. This scoring system, demonstrated favorable performance in both external cohort and conservative treatment cohort, integrates four clinical variables: ventricular system involvement, venous aneurysm, deep location, and exclusively deep drainage. Their findings also confirmed a significant association between deep location and an increased risk of hemorrhage in AVM patients. Our results support their findings; deep AVM location significantly increased subsequent hemorrhage risk (approximately four-fold). However, conflicting findings have been reported by Crawford et al. (27) who found that the temporal AVM location, instead of deep location, predicted a higher rupture rate. Due to the complexity of AVMs and the variety of classifications, the relationship between AVM location and subsequent hemorrhage needs to be further verified. Although exclusively deep venous drainage was associated with increased risk of subsequent hemorrhage in univariate analysis in this study, the association was not found to be significant in multivariate analyses. Based on the hemodynamic risk factors identified in this study, we believe that the increased intra-malformation pressure caused by inadequate venous drainage may be the primary cause of AVM rupture, while exclusively deep venous drainage alone may not significantly contribute to this stagnation.

Liew et al. (28) reported that spontaneous obliteration of an untreated AVM has an incidence rate of approximately 0.014% per year. However, even in patients with all features predictive of spontaneous obliteration, the expectation of achieving this when conservatively managed is not justified, and patients should expect a lifelong risk of subsequent hemorrhage from untreated AVMs. Spontaneous obliteration of an untreated AVM was not observed in any patient in the present study, which may be owing to the relatively short follow-up period and the fact that not every patient received an angiographic follow-up. Based on the extremely low spontaneous obliteration rate reported in previous literature, we believe that careful screening of AVMs at risk of subsequent hemorrhage is required to provide patients with the appropriate treatment.

### Limitations

Our study has several limitations, including its small sample size and relatively short follow-up period after DSA diagnosis. In addition, because of the overlap of the cerebrovascular system, our study only examined hemodynamic features in two dimensions, and measurements may have been vulnerable to inaccuracy. True visualization of three-dimensional DSA and larger studies with a longer follow-up period may be a future direction for exploring the precise hemodynamics of AVMs. Additionally, when comparing older ruptured AVMs with recently ruptured ones, potential bias may arise due to the potential differences in their subsequent hemorrhage risks. Ruptures could potentially alter the vascular structure within the nidus, leading to potential differences in hemodynamic features when comparing previously ruptured AVMs to unruptured ones. In forthcoming investigations with larger cohorts, including solely patients with unruptured AVM for the analysis of subsequent ruptures can help to eliminate this bias.

## Conclusion

A higher CBV and stasis index in the nidus, hemorrhagic presentation, and deep AVM location appear to be linked to a higher risk of subsequent hemorrhage in patients with untreated AVM. Hemodynamic features may help identify which patients will benefit from interventional therapy, assisting clinical decision-making for the management of AVMs.

## Data availability statement

The raw data supporting the conclusions of this article will be made available by the authors, without undue reservation.

## Ethics statement

The studies involving human participants were reviewed and approved by Beijing Tiantan Hospital. Written informed consent to participate in this study was provided by the participants’ legal guardian/next of kin.

## Author contributions

Material preparation, data collection, and analysis were performed by HZ and LL. Formal analysis and investigation were performed by YC, YS, and SL. CM, LZ, FL, and YZ performed manuscript review and editing. CJ performed funding acquisition and supervision. HZ wrote the first draft of the manuscript, and all authors commented on previous versions of the manuscript. All authors contributed to the article and approved the submitted version.

## Funding

This work was supported by the Beijing Natural Science Foundation project [grant number 7212007].

## Conflict of interest

The authors declare that the research was conducted in the absence of any commercial or financial relationships that could be construed as a potential conflict of interest.

The reviewers LM and YC declared a shared affiliation with the authors HZ, LL, YS, YC, LZ, CJ, and YZ to the handling editor at the time of review.

## Publisher’s note

All claims expressed in this article are solely those of the authors and do not necessarily represent those of their affiliated organizations, or those of the publisher, the editors and the reviewers. Any product that may be evaluated in this article, or claim that may be made by its manufacturer, is not guaranteed or endorsed by the publisher.
